# How to Use Not-Always-Reliable Binding Site Information in Protein-Protein Docking Prediction

**DOI:** 10.1371/journal.pone.0075936

**Published:** 2013-10-04

**Authors:** Lin Li, Yanzhao Huang, Yi Xiao

**Affiliations:** 1 Biomolecular Physics and Modeling Group, Department of Physics, Huazhong University of Science and Technology, Wuhan, Hubei, China; 2 Computational Biophysics and Bioinformatics, Department of Physics, Clemson University, South Carolina, United States of America; Università degli Studi di Milano, Italy

## Abstract

In many protein-protein docking algorithms, binding site information is used to help predicting the protein complex structures. Using correct and accurate binding site information can increase protein-protein docking success rate significantly. On the other hand, using wrong binding sites information should lead to a failed prediction, or, at least decrease the success rate. Recently, various successful theoretical methods have been proposed to predict the binding sites of proteins. However, the predicted binding site information is not always reliable, sometimes wrong binding site information could be given. Hence there is a high risk to use the predicted binding site information in current docking algorithms. In this paper, a softly restricting method (SRM) is developed to solve this problem. By utilizing predicted binding site information in a proper way, the SRM algorithm is sensitive to the correct binding site information but insensitive to wrong information, which decreases the risk of using predicted binding site information. This SRM is tested on benchmark 3.0 using purely predicted binding site information. The result shows that when the predicted information is correct, SRM increases the success rate significantly; however, even if the predicted information is completely wrong, SRM only decreases success rate slightly, which indicates that the SRM is suitable for utilizing predicted binding site information.

## Introduction

Most proteins interact with other proteins or molecules to perform their biological functions. On average, each protein interacts with three to ten partners approximately [1]. The details of protein-protein interactions need 3D structures of complexes. However, it is difficult to determine the structures of protein complexes experimentally, thus the number of available complex structures is still limited, compared with monomer protein structures. Therefore, it is helpful to use computational approaches to predict structures of protein complexes.

Many great docking algorithms have been developed. Some algorithms are based on Fast Fourier Transform (FFT) methods [2], such as MolFit [3], 3D-Dock [4], [5], [6], GRAMM [7], ZDock [8], [9], DOT [10], BiGGER [11], HEX [12] and so on. These FFT-based algorithms search 6D space fast and effectively. Thus, they are usually used as initial stages in docking procedures. However, the FFT-based algorithms consider receptor and ligand as rigid bodies. So, many of them are combined with other methods to further refine or re-rank the structures obtained in the initial stage [4], [13], [14]. Besides these FFT-based algorithms, some other algorithms are also developed, which are able to consider flexibility of proteins during docking procedure, such as RosettaDock [15], ICM-DISC [16], AutoDock [17], and HADDOCK [18].

If binding sites of a protein are known, they can be used to improve success rate of docking prediction [5], [19]. Many properties have been used to predict protein binding sites or interface residues and the widely used features include the hydrophobicity of residues [20], [21], [22], [23], the evolution conservation of residues [24], [25], [26], [27], [28], [29], planarity and accessible surface area of patches [30], [31]. Besides, some other interface-distinguishing features have also been explored. For example, it was found that the protein binding sites are surrounded by more bound waters and have lower temperature β-factors than other surface residues [32]. Some analysis also showed that protein interfaces are likely to contain backbone hydrogen bonds which are wrapped by more than nine hydrophobic groups [33]. Another work indicated that the side chains of interface residues have higher energies than other surface residues [34]. A single feature mentioned above cannot distinguish the binding sites from other surface residues. Thus some algorithms and meta servers have been developed, which combine different features to improve the binding site prediction success rate [32], [35], [36], [37], [38], [39], [40], [41]. A test on a dataset of 62 complexes shows that the success rates of these methods are about 30 percent [41].

Several groups integrate experimentally determined binding sites into their docking algorithms [4], [5], [19], [41], [42], [43], [44], [45]. These algorithms use the information in three different ways: (1) Most groups treat the information as a post filtering stage [4], [5], [41], [44], [45]. (2) Some algorithms [46], [47], [48], including Zdock’s block method [46], use the information to restrict the docking area during sampling stage. (3) Ben-zeev and Eisenstein implemented a weighted geometric method into Molfit [19]. For the first two kinds of algorithms, using correct binding site information can increase the success rate significantly, but obviously using wrong information will lead to a failed prediction. The third kind of algorithm could tolerant some inaccurate information, which made a success on a dataset of five complexes.

The predicted binding site information is not always reliable [41]. Thus, there is a high risk of using the unreliable information. In this work, A softly restricting method (SRM) is developed to utilize the predicted information. This SRM is based on our ASPDock algorithm [49], which has been proved to be successful in CAPRI(Critical Assessment of PRediction of Interactions) [50] rounds 18 and 19. SRM softly constrains the receptor and ligand to bind around predicted key residues during the sampling stage. The result shows that using SRM, the hit count number of the dataset increases significantly, which should greatly help scorers to pick out the near-native structures.

This work is different from Ben-zeev and Eisenstein’s. Ben-zeev and Eisenstein’s method is based on geometric complementary. On the contrast, our softly restricting method (SRM) is based on the ASPDock algorithm, which uses atomic solvation parameters (ASP) [51] rather than geometric complementary. Ben-zeev and Eisenstein test their method on several systems with experimental biochemical and biophysical data, which is correct information. However, in this work, we perform a large test on 99 complexes in benchmark 3.0 using only purely predicted information, which is mixed with correct and incorrect information.

## Results and Discussion

### Antibody-antigen and Dockground Complexes

**Figure 1:**
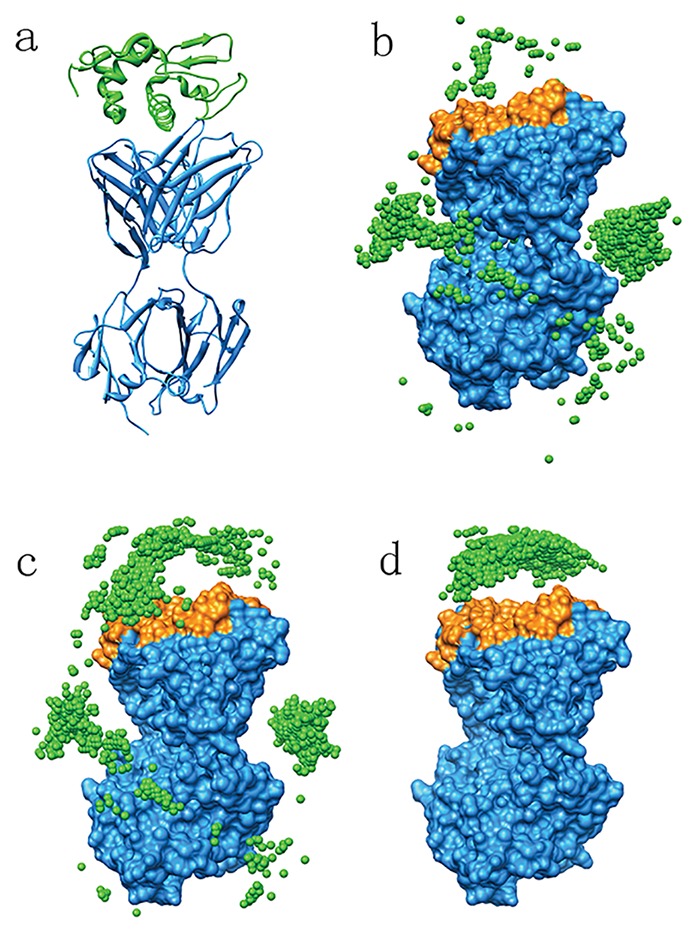
An example of antibody-antigen prediction. a. Native structure of antibody-antigen (1dqj), light blue structure is the receptor, Fab structure of antibody, CDR is colored orange. Green structure is the ligand. b. Ligand mass centers predicted by ASPDock without any predicted information. c. Ligand mass centers predicted by SRM, weight of CDR is 1.5. d. Ligand mass centers predicted by SRM, weight of CDR is 3.

**Figure 2:**
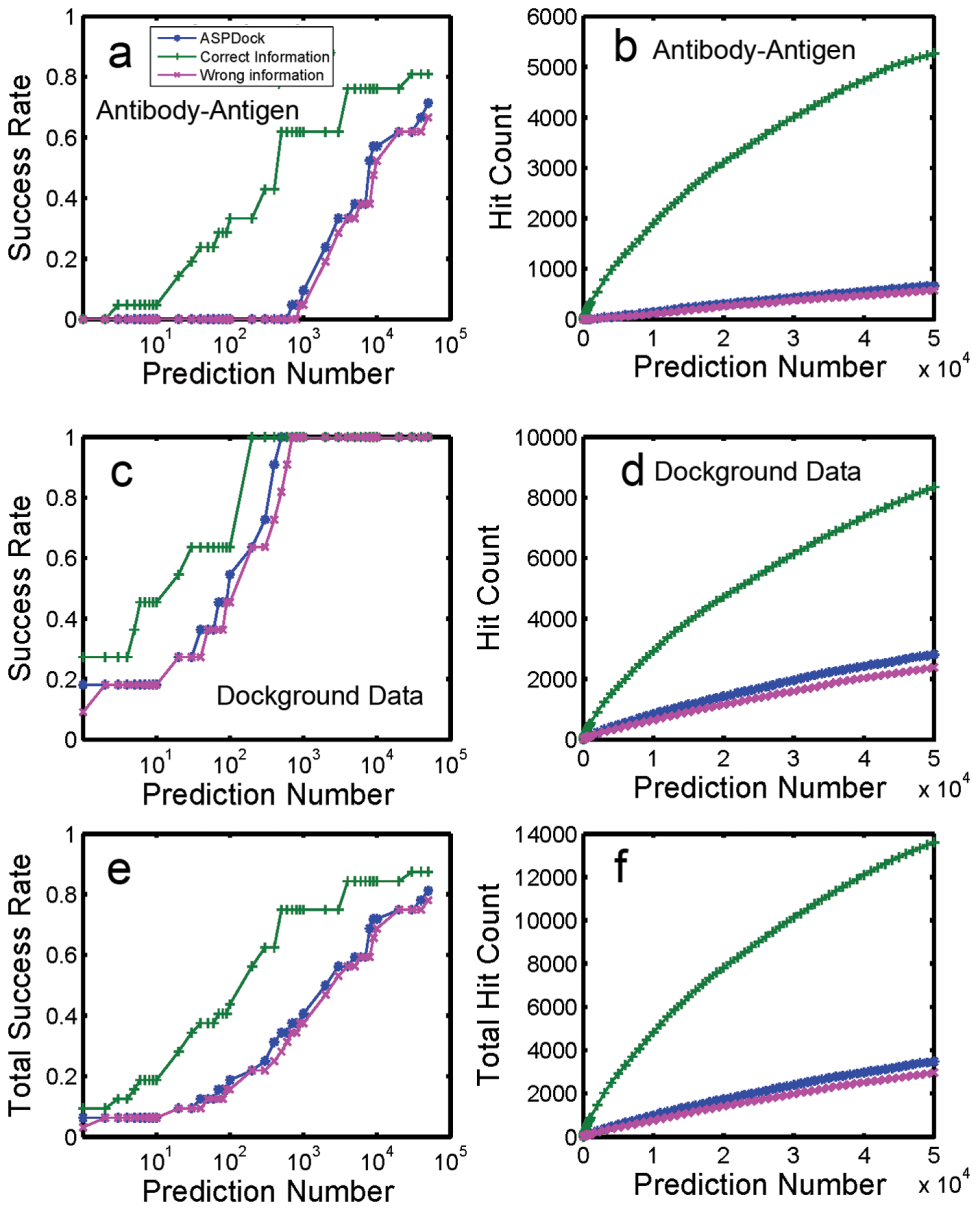
Results of 21 antibody-antigen and 11 dockground complexes.Predicted by ASPDock, SRM+Correct binding site information and SRM+Wrong binding site information. a. Success rate of antibody-antigens. b. Hit count of antibody-antigens. c. Success rate of Dockground complexes. d. Hit count of Dockground complexes. e. Success rate of total complexes. f. Hit count of total complexes.

Antibody-antigen complex structures are difficult to predict using ordinary FFT docking method without binding site information, mainly because each antibody Fab structure has two big pockets that are not the binding sites (Figure 1). The native binding site, CDR, usually has no advantage on geometry features. Using our ASPDock, antigens also have strong tendency to bind at the big pockets of antibodies because the accessible surface area decreases dramatically when antigens bind at the pockets. However, there are several methods to specify the CDR residues from sequences of antibodies. Using AbM definition, we specified CDR residues of all the 21 antibodies as correct information. We softly restrict the antigens to bind at the CDR residues and adjust the key residues weight in our algorithm by verifying the value of the weight factor α. When α>1.5, antigens strongly tend to bind at CDR residues. Consequently, the success rate and hit count are enhanced dramatically (Figures 2a and 2b).

However, even using correct information, there are still 5 antibody-antigen complexes that cannot be successfully predicted in top 2000 structures (Table 1), mainly because each of these complexes has a very small relative interface. In the top 2000 predictions, these 5 antigens tend to bind around the CDR residues of their conjunct antibodies, but the predicted interfaces of antigens are not correct. It implies that for these 5 antibody-antigen complexes, only information of CDR cannot make a successful prediction and it also needs to know antigen’s binding sites (antigenic determinant).

**Table 1:** Results of antibody-antigen and dockground complexes predicted by ASPDock and SRM.

				ASPDock					SRM					
PDB	HitCount		FirstRMSD(Å)	FirstRank	BestRMSD(Å)	BestRank	HitCount	FirstRMSD(Å)	FirstRank	BestRMSD(Å)	BestRank	RelativeInterfacearea	UB-RMSDofreceptor (Å)	UB-RMSDofligand(Å)
Antibody-antigen														
1ahw		0	–	–	–	–	8	5.52	463	2.37	936	0.07	0.7	1.38
1bgx		0	–	–	–	–	0	–	–	–	–	0.11	1.55	1.34
1bj1		0	–	–	–	–	0	–	–	–	–	0.06	0	0.72
1bvk		3	6.82	1043	6.37	1896	60	7.25	16	5.62	1670	0.08	0.81	1.16
1dqj		0	–	–	–	–	3	9.52	828	9.37	1137	0.07	0.79	0.82
1e6j		0	–	–	–	–	24	9.58	243	3.84	1946	0.04	1.11	1.54
1fsk		0	–	–	–	–	57	3.31	203	3.22	756	0.06	0	0.59
1i9r		0	–	–	–	–	6	9.57	142	6.74	422	0.04	1.5	0
1iqd		3	4.08	948	3.87	1716	65	3.35	3	3.02	1308	0.08	0	0.68
1jps		7	8.07	602	8	1036	37	8.5	27	2.56	104	0.07	0.68	1.01
1k4c		0	–	–	–	–	0	–	–	–	–	0.07	0	0.6
1mlc		0	–	–	–	–	52	5.96	259	4.95	434	0.06	1.05	0.74
1nby		0	–	–	–	–	1	9.19	1255	9.19	1255	0.07	0.8	0.79
1nca		0	–	–	–	–	28	5.17	91	1.5	462	0.06	0	0.23
1nsn		2	3.24	1691	3.24	1691	59	3.28	64	1.59	1071	0.07	0	0.76
1vfb		0	–	–	–	–	63	6.9	36	3.4	543	0.09	0.56	0.98
1wej		0	–	–	–	–	2	9.92	145	9.92	145	0.05	0.9	0.4
2fd6		0	–	–	–	–	10	7.85	472	7.74	1183	0.04	1.24	3.48
2hmi		0	–	–	–	–	0	–	–	–	–	0.02	3.54	0
2jel		3	9.01	1447	7.76	1501	64	7.76	14	5.16	134	0.07	0	0.76
2vis		0	–	–	–	–	0	–	–	–	–	0.04	5.4	0.59
Dockground														
1a2y		3	7.37	280	7.01	1822	2	7.37	388	7.05	1260	0.08	0.66	1.27
1cgj		84	2.7	1	1.88	291	64	2.7	1	1.88	406	0.16	0.35	1.14
1cse		6	9.34	496	9.25	1765	6	9.34	690	9.34	690	0.12	0.30	1.19
1f7z		7	9.53	378	9.27	1803	5	9.53	554	9.28	573	0.11	0.32	0.28
1ppf		39	9.35	63	5.92	1447	30	9.35	88	6.74	1966	0.1	0.38	0.45
1shw		25	6.84	13	5.73	1933	19	6.84	20	5.98	128	0.07	2.75	0.87
1tx4		25	7.47	1	5.02	647	21	7.47	2	5.02	1044	0.13	0.67	0.41
1uex		5	7.15	154	7.05	1399	4	7.15	170	7.05	1585	0.09	0.58	0.43
2jb0		11	8.15	92	6.23	592	10	8.15	133	6.23	840	0.12	0.48	0.52
2kai		20	9.16	325	6.95	683	13	9.16	437	6.95	921	0.11	0.68	0.54
2pav		25	5.52	35	3.66	878	22	5.52	48	3.66	1144	0.11	1.11	0.76

Hit count and success rate are analyzed form top 2000 predictions of each complex. Relative interface area, UB-RMSD of receptors and ligands implicate the difficulty of prediction.

The sensitivity of SRM to incorrect information is also tested. For each antibody, we randomly selected 10 surface but non-interface residues as incorrect information. All the incorrect residues are out of CDR biding site, therefore, the incorrect information should result decrease of success rate and hit count. When the incorrect information is used for these 21 antibody-antigen complexes and the weight factor α is still set as1.5, success rate and hit count decrease slightly. This indicates that SRM is insensitive to incorrect information (Figures 2a and 2b).

For test on the 11 dockground3.0 complexes, when the weight factor α is also set as 1.5, success rate and hit count increase for correct information and did not decrease significantly for incorrect information (Figures 2c and 2d). This indicates that ASPDock evaluates near-native predictions as high score predictions, which are easy to get into top rank when the weight factor is 1.5. By contrast, most wrong predictions are evaluated as low score structures, even the ASP values of their binding site residues are enhanced 1.5 times, they still have no enough high scores to get into top rank.

### Enzyme-inhibitor and Other Complexes

**Figure 3:**
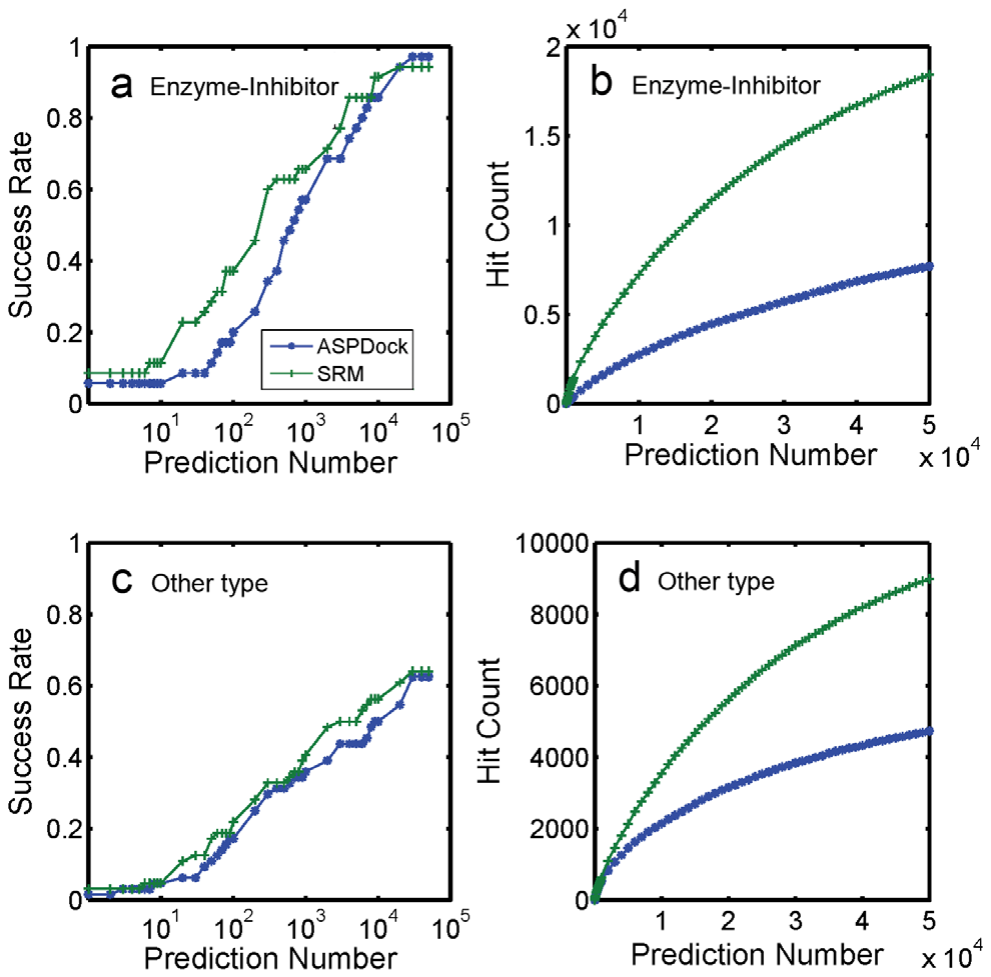
Results of 35 enzyme-inhibitor complexes and 64 other type complexes. Predicted by ASPDock and SRM. a. Success rate of enzyme-inhibitors. b. Hit count of enzyme-inhibitors. c. Success rate of other complexes. d. Hit count of other complexes.

The tests on 21 antibody-antigen and 11 dockground3.0 complexes demonstrate that using SRM, correct information improves success rate and hit count significantly, while the incorrect information reduces success rate and hit count only slightly (Figures 2e and 2f). This means SRM is suitable for utilizing predicted information. Therefore, we test SRM on a 99-complexes data set by using predicted information from PPI-PRED server (figure 3).

This data set includes 35 enzyme-inhibitor complexes and 64 other type complexes. For enzyme-inhibitor complexes, ASPDock has already made a high success rate without using any predicted information, 24 out of 35 complexes are successfully predicted (in top 2000 predictions). Using information provided by PPI-PRED, the success rate doesn’t increase significantly, and 25 out of 35 complexes were successfully predicted (in top 2000 predictions). However, the hit count number in top 2000 predictions increases from 742 to 2348 (Table 2). This improvement could help scorers easier to pick up the near-native structures using their scoring functions.

**Table 2:** Results of enzyme-inhibitor and other complexes predicted by ASPDock and SRM.

			ASPDock				SRM					RelativeInterfacearea				UB-RMSDofreceptor (Å)				UB-RMSDofligand (Å)						
PDB	Accuracy-R^a^(%)	Accuracy-L^a^(%)	Hit Count	BestRMSD (Å)	Firstrank	HitCount		BestRMSD (Å)			Firstrank															
enzyme-inhibitor																										
1acb	41.18	14.29	3	8.50	168		7			7.77	301				0.12				1.61				1.32			
1avx	36.67	16.00	0	–	–		7			9.73	72				0.10				0.46				0.52			
1ay7	40.00	34.62	22	2.29	335		233			1.28	11				0.13				0.46				0.55			
1bvn	34.37	86.67	60	2.56	60		163			1.94	43				0.11				0.58				0.36			
1cgi	43.14	44.44	23	6.06	451		117			2.29	79				0.16				1.33				1.52			
1d6r	6.98	38.46	11	7.71	67		7			8.66	125				0.12				1.08				0.94			
1dfj	62.07	19.35	1	8.58	1184		50			2.33	7				0.11				0.65				1.50			
1e6e	6.10	29.63	43	3.26	111		24			3.88	196				0.09				1.04				1.05			
1eaw	47.22	73.33	20	3.92	459		233			2.56	14				0.15				0.53				0.48			
1ewy	26.09	53.85	94	3.14	12		212			2.78	1				0.08				1.00				0.76			
1ezu	40.00	15.69	0	–	–		0			–	–				0.12				0.33				2.21			
1f34	22.92	42.11	8	5.35	300		10			5.21	198				0.16				0.61				1.09			
1fq1	3.70	2.56	0	–	–		0			–	–				0.08				0.72				3.19			
1hia	–	55.56	53	7.48	1		115			7.42	1				0.14				0.78				1.96			
1ijk	0.00	0.00	0	–	–		0			–	–				0.08				0.88				0.43			
1kkl	0.00	47.62	0	–	–		1			8.68	1718				0.06				2.84				0.43			
1m10	40.62	0.00	0	–	–		0			–	–				0.10				1.22				1.66			
1mah	28.12	24.00	1	8.35	1273		2			2.58	1732				0.10				0.73				0.64			
1n8o	85.71	45.71	1	4.10	861		55			2.14	16				0.12				0.49				0.77			
1nw9	30.95	0.00	7	7.37	49		16			5.38	37				0.14				2.83				0.66			
1oph	0.00	40.00	0	–	–		0			–	–				0.06				1.52				0.31			
1ppe	42.11	50.00	310	1.64	1		461			1.27	1				0.17				0.42				0.40			
1pxv	11.76	36.11	0	–	–		0			–	–				0.15				2.41				0.81			
1r0r	45.00	50.00	13	2.13	243		132			1.86	205				0.12				0.31				0.60			
1tmq	31.82	41.18	5	3.10	437		72			2.13	55				0.12				0.38				0.91			
1udi	44.44	76.19	28	3.40	234		248			2.21	13				0.15				0.45				0.92			
1yvb	38.10	28.57	11	4.15	577		93			2.85	285				0.10				0.58				2.62			
2b42	4.35	18.87	0	–	–		0			–	–				0.12				0.72				0.70			
2mta	0.00	38.89	12	7.28	98		19			7.28	248				0.06				0.44				0.58			
2o8v	21.15	11.11	0	–	–		0			–	–				0.10				1.02				1.20			
2pcc	20.63	33.33	0	–	–		15			6.19	797				0.06				0.36				0.48			
2sic	31.43	0.00	1	6.58	1151		0			–	–				0.11				0.27				0.61			
2sni	43.75	56.25	7	6.92	775		26			7.66	223				0.13				0.26				0.42			
2uuy	0.00	36.00	1	9.55	1761		0			–	–				0.11				0.31				1.85			
7cei	42.11	17.39	7	4.22	634		30			4.93	279				0.12				1.10				1.60			
other																										
1a2k	13.95	16.13	0	–	–		0		–		–			0.08				1.11				1.10				
1ak4	41.38	0.00	0	–	–		0		–		–			0.07				0.52				1.36				
1akj	0.00	4.00	21	6.00	78		8		7.26		830			0.07				1.14				0.89				
1atn	0.00	0.00	0	–	–		0		–		–			0.07				2.64				0.43				
1azs	0.00	29.51	0	–	–		0		–		–			0.06				0.00				0.51				
1b6c	56.00	13.79	4	6.46	555		28		2.87		132			0.09				0.31				1.82				
1bkd	0.00	0.00	0	–	–		0		–		–			0.11				2.42				3.12				
1buh	2.08	40.91	36	3.27	52		2		2.88		1578			0.08				1.02				1.02				
1de4	0.00	0.00	0	–	–		0		–		–			0.03				1.31				1.61				
1e96	27.27	30.00	3	8.45	208		14		6.10		128			0.07				0.68				0.59				
1eer	58.82	0.00	0	–	–		14		7.66		50			0.11				3.79				3.75				
1efn	9.52	28.00	1	9.74	1627		5		6.94		612			0.15				0.56				0.59				
1f51	0.00	40.74	27	2.21	190		18		4.63		281			0.11				1.37				0.70				
1fak	0.00	19.05	0	–	–		0		–		–			0.14				6.09				1.67				
1fc2	40.00	3.92	0	–	–		0		–		–			0.09				0.00				0.80				
1fqj	37.74	43.75	2	6.74	1414		69		4.36		26			0.08				0.51				0.85				
1gcq	0.00	52.00	1	8.90	697		83		6.50		201			0.17				0.58				1.01				
1ghq	0.00	10.34	0	–	–		0		–		–			0.04				1.02				0.70				
1gla	0.00	25.00	0	–	–		0		–		–			0.05				0.61				0.37				
1gp2	19.05	0.00	0	–	–		0		–		–			0.07				1.62				1.52				
1gpw	0.00	44.44	11	4.99	119		71		1.15		19			0.11				3.44				0.60				
1grn	10.53	4.26	0	–	–		14		3.88		130			0.13				1.63				0.57				
1h1v	15.71	0.00	0	–	–		0		–		–			0.07				1.50				13.90				
1he1	6.06	3.23	32	5.72	20		29		5.48		16			0.15				0.83				0.71				
1he8	0.00	0.00	0	–	–		0		–		–			0.03				1.57				0.60				
1i2m	48.84	26.04	0	–	–		9		5.86		1037			0.13				2.45				1.03				
1i4d	9.09	15.00	0	–	–		0		–		–			0.06				0.88				1.26				
1ib1	0.00	48.48	0	–	–		0		–		–			0.10				2.29				0.62				
1ibr	41.86	21.21	0	–	–		0		–		–			0.12				0.00				2.91				
1ira	42.03	86.21	0	–	–		0		–		–			0.15				19.58				0.59				
1j2j	29.73	29.73	247	2.42	32		64		2.06		154			0.11				1.08				1.02				
1jmo	7.14	0.00	0	–	–		1		6.39		1982			0.11				3.69				0.44				
1k5d	0.00	48.08	0	–	–		0		–		–			0.08				1.54				0.73				
1k74	0.00	42.55	40	3.53	67		121		1.60		1			0.09				1.01				1.44				
1kac	5.71	41.67	2	9.48	1697		0		–		–			0.10				0.48				0.91				
1klu	0.00	45.71	0	–	–		0		–		–			0.04				1.27				0.96				
1ktz	0.00	0.00	0	–	–		0		–		–			0.09				2.03				0.60				
1kxp	30.00	18.88	40	2.75	41		63		2.20		1			0.09				0.81				2.09				
1ml0	0.00	75.00	0	–	–		41		2.79		41			0.06				1.52				1.25				
1n2c	0.00	0.00	0	–	–		0		–		–			0.05				0.43				4.02				
1qa9	3.33	0.00	0	–	–		0		–		–			0.12				0.76				0.66				
1r8s	59.37	79.31	0	–	–		0		–		–			0.18				3.89				1.31				
1rlb	0.00	0.00	3	8.36	190		0		–		–			0.05				0.70				0.51				
1s1q	17.24	50.00	33	2.42	260		21		2.26		538			0.11				0.70				1.01				
1sbb	16.67	0.00	0	–	–		0		–		–			0.05				0.89				0.49				
1t6b	1.32	0.00	0	–	–		0		–		–			0.05				1.43				1.07				
1wq1	0.00	0.00	15	7.47	245		8		6.00		803			0.14				0.93				0.91				
1xd3	41.18	87.50	63	5.12	40		122		4.66		55			0.17				1.05				0.83				
1xqs	28.77	7.29	0	–	–		1		9.94		1591			0.12				2.15				0.63				
1y64	0.00	0.00	0	–	–		0		–		–			0.07				10.28				0.95				
1z0k	19.35	50.00	96	2.17	8		50		2.39		14			0.17				0.90				0.41				
1z5y	0.00	11.11	28	4.85	84		0		–		–			0.10				1.02				0.98				
1zhi	18.18	0.00	0	–	–		0		–		–			0.07				1.22				1.73				
2ajf	1.02	0.00	0	–	–		0		–		–			0.05				0.46				2.83				
2btf	20.00	37.93	0	–	–		11		5.39		99			0.10				2.69				0.59				
2c0l	4.41	27.27	0	–	–		1		9.91		1842			0.11				1.78				3.89				
2cfh	43.75	41.67	123	3.27	1		94		2.74		6			0.15				1.05				0.00				
2h7v	0.00	0.00	0	–	–		0		–		–			0.07				1.76				1.13				
2hle	27.91	41.46	54	3.19	3		54		3.21		14			0.14				1.89				0.84				
2hqs	7.14	0.00	26	7.49	183		0		–		–			0.11				2.41				0.55				
2hrk	39.13	30.43	0	–	–		3		5.02		923			0.11				0.99				0.84				
2nz8	0.00	0.00	37	6.88	141		29		6.06		44			0.11				2.37				1.80				
2oob	66.67	62.50	3	7.76	956		39		2.16		93			0.12				0.65				0.83				
2ot3	83.33	80.95	2	9.40	334		7		8.97		201			0.12				1.09				2.69				

a Accuracy here is calculated by Nsuc/Npred, which is mentioned in method section.

Hit count and success rate are analyzed form top 2000 predictions of each complex. Relative interface area, UB-RMSD of receptors and ligands implicate the difficulty of prediction.

For 64 complexes of other types, ASPDock successfully predicts 26 complexes in top 2000 predictions. This number increases to 31 (by 19%) by using SRM with binding site information from PPI-PRED. However, hit count in top 2000 doesn’t increase a lot, which is raised from 831 to 1094.

As a first stage sampling algorithm, the most important goal is obtaining as many hits as possible. For all of the 99 complexes, the number of correctly predicted complexes from ASPDock is 50, total hit count from ASPDock is 1573, and thus the average hit count for ASPDock is 31.5; By contrast, the number of correctly predicted complexes from SRM is 56, total hit count from SRM is 3442, therefore the average hit count for SRM is 61.5. Once more, it demonstrates that SRM is able to get a better success rate as well as larger average hit count. Here we noticed that the average hit count from SRM is increased to almost twice as from ASPDock, which is very useful for the scoring functions to pick up the correct structures from the top 2000 structures for each complex.

In the above results, all the hits are defined as structures with LRMSD≤10 Å, which are “acceptable predictions” in CAPRI criterion. In order to test how SRM performs on predicting “medium predictions”, we did another analysis by defining hits to be structures with LRMSD≤5 Å. Under this definition, For all of the 99 complexes, the number of correctly predicted complexes from ASPDock is 23, total hit count from ASPDock is 284, and thus the average hit count for ASPDock is 12.3; By contrast, the number of correctly predicted complexes from SRM is 31, total hit count from SRM is 834, therefore the average hit count for SRM is 26.9. This analysis indicates that even the criterion is stricter, the SRM still works better than ASPDock. We didn’t test the performance of SRM on predicting “high accuracy predictions” (LRMSD≤2.5 Å). Because without scoring function and structure refinement program, SRM, a sampling stage algorithm, is not supposed to be good at obtaining “high accuracy predictions”.

As mentioned in method section, the weight factor α value is searched from 1.0 to 3.0 by a step of 0.1, and we found the optimized value of α is 1.5, which can enhance the success rate when using correct information and tolerate some incorrect information. The weight factor α is the key parameter, it effects the success rate and hit count. For example, when the α is set as 2.0 and the criterion for hit is set as LRMSD≤10 Å, the number of correctly predicted complexes from SRM is 53, total hit count from SRM is 3051, therefore the average hit count for SRM is 57.6. The reason for the decrease is that when the α value is enhanced, the wrong information gets more weight, which may decrease the success rate. However, the optimized weight factor equal to 1.5 is based on the atomic solvation parameters scoring function in ASPDock. Other docking method based on different scoring functions may need different optimized weight factors.

The results on 21 antibody-antigen complexes and 11 dockground3.0 complexes demonstrate that by using proper weight factor, our protein-protein docking sampling method is sensitive to correct information and insensitive to incorrect information. Based on this feature, we only use purely predicted information to test 99 complexes in benchmark3.0. The result shows that the SRM can improve docking prediction significantly, even when the information used is not totally correct.

## Conclusions

Results on antibody-antigen and dockground 3.0 complexes indicate that SRM is much more sensitive to correct information than wrong information. This implies that SRM is effective if we know all or some of the native binding sites. Moreover, SRM can tolerate some wrong information. Results on enzyme-inhibitor and other complexes show that using predicted information overall hit count number increases significantly and success rate is also raised. The result should be better if predicted information is more accurate.

In our test on 99 complexes from benchmark3.0, only purely theoretically predicted information is used. Currently, there are lot of great works focusing on enhancing the success rate of theoretical binding site prediction. It is believed that the theoretical binding site prediction method will be more accurate in the future due to those great works. We will keep on improving our SRM to utilize the theoretically predicted binding information more effectively. Combining the binding site prediction method and protein-protein docking method together to predict the protein-protein interaction should be more widely used in the future.

## Methods

### ASPDock

ASPDock is a docking algorithm based on FFT method [49]. Traditional FFT docking methods consider the shape complementarity as a crucial criterion to rank the predicted complex structures [2]. ASPDock implements atomic solvation parameters in traditional FFT method to rank the predicted complex structures. ASPDock performs better than the shape complementarity docking method on benchmark3.0 [52], and it also made successes in CAPRI rounds 18 and 19.

In ASPDock [49], receptor and ligand are projected on 3-dimensional grids as follows:
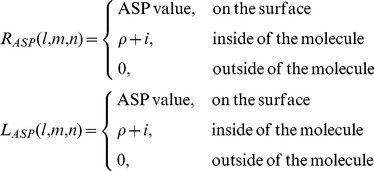
(1)


ASP (atomic solvation parameters) value here depends on atom type, which is always a negative number. 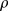 is a constant positive number, which is a penalty for protein-protein overlap. In this work 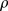 = 20. 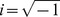 is the imaginary unit.

Then we can search the 3-dimensional translation space by calculating the correlation function:
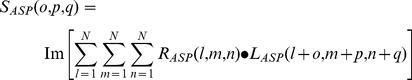
(2)


This calculation can be accelerated by using FFT method,
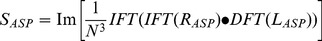
(3)


For rotation scan, we use 10 degree step and pick up top 3 structures in each rotation. Grid step in translation scan is 1 Å.

## Softly Restricted Method

Based on the ASPDock [49], we develop a softly restricting method (SRM) to utilize the predicted binding site information. The residues at the predicted binding sites are taken as key residues. We enhance the ASP value of these key residues by multiplying a weight factor α, and keep ASP values of other residues unchanged.
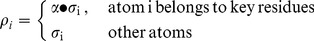
(4)where 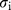 is the original ASP value and 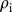 is the enhanced ASP value of atom i. α>1 if atom i is expected to be on the interface. 0<α<1 if atom i is expected to be NOT on the interface. In this work, we don’t consider the later situation.

Then based on ASPDock, we can search the 6-dimensional space using 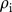 instead of 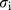 and pick up top N predictions. These N predictions should tend to bind at the key residues. The tendency could be adjusted by the weight factor α, and a larger α leads to a stronger tendency to bind at the key residues.

As shown by Huang in 2008 [41], success rate of predicting interface residues is only about 30%, there is a risk to use predicted information. Thus the weight factor α should be a moderate value and it cannot be a very large number. In this work a simple grid step method is used to optimize the weight factor α. We search α value from 1 to 3 by a step of 0.1, and found the optimized value of α is 1.5, which can enhance the success rate when using correct information and tolerate some incorrect information.

## Dataset

Most docking algorithms can improve the predictions if correct information is used. However, if the information is incorrect, the post filtering algorithms and restrict algorithms would fail to predict near-native structure. Predicted information cannot be always correct. When using the predicted information, the crucial problem is to keep docking success rate not decreasing when information is incorrect.

In this work, 21 antibody-antigen complexes from benchmark3.0 [52] and dockground3.0 [53] are selected as our training set. Totally there are 30 non-redundant antibody-antigen complexes in benchmark3.0 and dockground3.0. For these antibody-antigen complexes, we only select the complexes that contain the entire Fab (Fragment of antigen binding region) structures. Because the complexes with entire Fab structures are difficult for docking programs without any information and their complementarity determining regions (CDR) could be detected by AbM definition or other prediction methods. Thus 9 out of 30 complexes are removed from our training set. Antibody proteins with Fab structures are well studied and their binding sites can be easily specified from their sequences. There are several different methods (http://www.bioinf.org.uk/abs/) to specify the CDR of antibodies. Here we use a simple method of AbM definition (http://www.bioinf.org.uk/abs/). The results have no significant change if we choose other methods. As the binding site of antibodies could be well predicted before docking, the antibody-antigen training set is suitable for assessing the SRM’s ability to use correct predicted information during docking procedure. We also randomly selected 10 surface but non-interface residues for each antibody as wrong information.

Antibody-antigen complexes are difficult to predict without predicted binding site information. Besides the antibody-antigen complexes, we also selected some other complexes which are easier to predict than antibody-antigen complexes. These complexes are selected from dockground3.0 rank1 and all of the bound-unbound complexes are removed. The redundant complexes compared to benchmark3.0 are also removed. After these filtering procedures, 17 complexes remain. Using our ASPDock, we successfully predicted (at least 1 hit in top 2000 predictions) 11 in 17 of these complexes. For each of these 11 complexes’ receptor, we randomly selected 10 interface residues as correct information and 10 surface but non-interface residues as incorrect information. Our training set is built up by these 11 complexes and 21 antibody-antigen complexes mentioned above with correct and incorrect information.

Enzyme-inhibitor and other type complexes of benchmark3.0 are selected as our test dataset. This test dataset totally contains 99 complexes, including 35 enzyme-inhibitor and 64 complexes of other types. We predicted the binding sites for each monomer in this dataset using PPI-PRED [37].

## PPI-PRED

Five binding site prediction methods have been test on a data set in Huang and schroeder’s work. Success rate of these methods are from 14 to 34 percents. Among the five methods, PPISP [40] and PPI-PRED [37] have 34% and 33% success rate, respectively. PPI-PRED considers more sequence and structure features than PPISP and is selected as the prediction method in our work.

## Criterion

LRMSD is the RMSD between the predicted and native ligand molecules after superposing the predicted and native receptor molecules. LRMSD is used as a criterion in CAPRI (Critical Assessment of PRediction of Interactions) [50]: predictions with LRMSD≤10 Å are considered as “acceptable predictions”; predictions with LRMSD≤5 Å are considered as “medium predictions”; predictions with LRMSD≤2.5 Å are considered as “high accuracy predictions”. This CAPRI style measure is widely used in protein-protein docking and scoring works. [14], [49], [54], [55]; In this work, a hit is defined as a predicted complex with LRMSD≤10 Å, which is an “acceptable prediction”. Since our SRM is a structure sampling method, which is the first stage of the entire docking algorithm, the LRMSD of acceptable structures could be decreased after some other refinement process. [56], [57], [58].

A residue is a surface residue if there is more than 10% relative residue surface area exposed to solvent, where the surface area is calculated by NACCESS (http://wolf.bms.umist.ac.uk/naccess). An interface residue is defined as a surface residue if the minimum distance of its atoms from the atoms of another protein in the native complex structure is less than 5 Å. We don’t use 10 Å as a criterion because it is useless if a predicted binding site is 10 Å away from interface. The radius of some small protein is no more than 20 Å. For each monomer, accuracy of prediction is calculated by 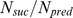. Here 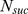 is the number of successful predicted interface residues, and 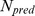 is total number of predicted interface residues.

We used unbound-bound RMSD (UB-RMSD) and relative interface area to assess the difficulty to predict each complex. UB-RMSD is the RMSD between unbound and bound monomers. Relative interface area is the ratio of interface area and total complex area. Obviously a complex is difficult to predict if it has a large UB-RMSD of its monomers, or if it has a small relative interface area.

Our SRM is a first stage sampling method, which should be combined with some post processing methods. [47], [48], [59], [60] Currently, most post processing methods are able to handle at least 2000 structures. [55], [61], [62] The post processing methods are aiming at re-score the top 2000 (or even more) predictions and then pick up the best 10–20 predictions. Thus, for each docking prediction, we keep top 2000 predicted structures for further analysis.
